# Association of Nonalcoholic Fatty Liver Disease With Osteoporotic Fractures: A Cross-Sectional Retrospective Study of Chinese Individuals

**DOI:** 10.3389/fendo.2018.00408

**Published:** 2018-07-23

**Authors:** Yanmao Wang, Gen Wen, Runhua Zhou, Wanrun Zhong, Shengdi Lu, Chengfang Hu, Yimin Chai

**Affiliations:** Department of Orthopedic Surgery, Shanghai Jiao Tong University Affiliated Sixth People's Hospital, Shanghai, China

**Keywords:** nonalcoholic fatty liver disease, fracture, cross-sectional studies, risk factor, dyslipidemia

## Abstract

**Objective:** Nonalcoholic fatty liver disease (NAFLD) is related to several inflammatory or metabolic diseases. However, findings of previous studies investigating the association between NAFLD and BMD are inconsistent. Only one study reported a potential association between NAFLD and osteoporotic fracture. This study investigated whether NAFLD in older participants (>55 years) was associated with osteoporotic fracture risk.

**Materials and Methods:** This cross-sectional, observational study included 2,695 participants (35.7% men, 614 cases of NAFLD, and 383 fractures). Standardized questionnaires, laboratory tests, and physical and ultrasonic examinations were completed.

**Results:** After adjusting for various factors including serum triglycerides (TG), high-density cholesterol (HDL-C), and low-density lipoprotein cholesterol (LDL-C), multivariate logistic regression models revealed a marginal association between NAFLD and osteoporotic fracture risk in men (odds ratio [OR], 1.86; 95% confidence interval [CI], 1.06–3.27; *P* = 0.030) but no association in women (OR, 1.05; 95% CI, 0.74–1.48; *P* = 0.800). Further stratified analyses showed a significant association between NAFLD and osteoporotic fracture risk in men without high TG, low HDL-C, and high LDL-C.

**Conclusions:** There was a significant association between NAFLD and osteoporotic fracture risk in older Chinese men, particularly men without dyslipidemia.

## Introduction

Osteoporosis is a systemic skeletal disease characterized by bone loss and bone microstructure degradation that leads to low energy fractures (1, 2). The incidence of osteoporosis and osteoporotic fractures is increasing, resulting in increased morbidity, increased mortality, and heavy burdens on families in terms of social status and economic status (1, 2). The risk of osteoporotic fractures is associated with many factors, including metabolic factors, inflammatory state, disease status, lifestyle, and genetic factors (3–5). Recently, the relationship between inflammation, metabolism, and osteoporosis has been elucidated (6, 7), and risk factors for osteoporotic fracture such as subclinical thyroid dysfunction, metabolic syndrome, and insulin resistance have been further explored (8, 9).

Nonalcoholic fatty liver disease (NAFLD), typically presents as mild or heavy steatosis, is the most common pathological disorder of the liver. It is associated with end-stage liver disease and liver-related morbidity and mortality (10). NAFLD is considered to be a multisystem disease that may affect many extrahepatic organs, is associated with various metabolic abnormalities, and can influence and participate in multiple metabolic pathways (10). Moreover, NAFLD is associated with several risk factors that affect bone mineral density (BMD), such as chronic inflammatory disease, metabolic syndrome, cardiovascular disease, diabetes mellitus, and chronic kidney disease (10, 11–13). However, findings of previous studies investigating the association between NAFLD and osteopenia/osteoporosis are inconsistent (14–17).

To our knowledge, there is only one cross-sectional study that has investigated the association between NAFLD and risk of osteoporotic fractures in adults aged ≥40 years. After adjusting for age, smoking, alcohol intake, physical activity, metabolic risk factors, kidney function, and use of glucocorticoids or osteoporosis medications, this study found that NAFLD may significantly increase the odds ratio of osteoporotic fracture risk in men (14). However, this study included both middle-aged and elderly participants; therefore, confounding factors, such as menopause and BMD, may have affected the real association between NAFLD and risk of osteoporotic fractures.

Therefore, the objectives of this study were to investigate whether NAFLD in elderly participants (aged >55 years) was associated with osteoporotic fracture risk and to further examine whether other factors may affect this association.

## Materials and methods

### Study population and design

This population-based, cross-sectional study recruited 3,657 participants (4,000 total participants and 343 dropouts) aged 55–85 years from the Shanghai metropolitan area in China between June 2014 and June 2016. This study was approved by the Ethics Committee of Shanghai Sixth People's Hospital, and all participants signed an informed consent form. The exclusion criteria were as follows: (1) history of hepatitis, liver cirrhosis, or any cancer or malignancy (*n* = 285); (2) history of kidney disease (*n* = 82); (3) regular treatment for osteoporosis (*n* = 192); (4) excessive alcohol consumption (≥140 g/week for men and ≥70 g/week for women; *n* = 311); and (5) absence of completed questionnaires (*n* = 128). Therefore, a total of 2,659 (950 men and 1,709 women) participants were included in the analysis (Figure S1). The study design was described in our previous article (15).

### Participant characteristics

Standardized self-administered questionnaires were used to collect participant characteristics such as demographic characteristics, lifestyle, disease history, and medication history. Chronic disease history, including cardiovascular disease, diabetes, hypertension, chronic kidney disease, dyslipidemia, and fractures, was also recorded. Smoking status was categorized as nonsmoker, current smoker, or ex-smoker. Alcohol consumption status was categorized as never, current (drinking within the past 6 months), or previous (cessation of more than 6 months). The type, frequency, and dose of alcohol consumption were collected and mean daily alcohol consumption was calculated. Physical activity was classified as <30 min per day, 30–60 min per day, 1–2 h per day, 2–4 h per day, 4–6 h per day, and >6 h per day.

Physical examination was performed according to a standardized protocol and by a trained inspector. Standing height and body weight were measured while participants wore lightweight clothing and no shoes. Systolic blood pressure and diastolic blood pressure were measured with an automatic sphygmomanometer after at least 5 min of seated rest. Body mass index (BMI), which was used as an index of body fat, was calculated as weight divided by height (kg/m^2^). Waist circumference was measured horizontally at the umbilicus level. Total hip BMD was measured by dual energy X-ray absorptiometry using a Lunar Prodigy GE densitometer (Lunar Corp, Madison, WI, USA).

Laboratory tests included triglycerides (TG), total cholesterol (TC), high-density lipoprotein cholesterol (HDL-C), and low-density lipoprotein cholesterol (LDL-C). All values were measured by an automated analyzer (Modular E170; F. Hoffmann-La Roche Ltd, Basel, Switzerland) using standard methods. Dyslipidemia was defined as having high TG (≥1.7 mmol/L), high TC (≥5.0 mmol/L), high LDL-C (≥2.6 mmol/L), and low HDL-C (≤0.9 mmol/L in men and ≤1.1 mmol/L in women) (16, 17).

### Definition of NAFLD

NAFLD was diagnosed based on the results of a liver ultrasonic examination. Two ultrasonographers examined all participants and were blinded to their clinical information. Fatty liver disease was diagnosed when characteristics of increased diffuse echogenicity, ultrasound beam attenuation, and poor visualization of intrahepatic architecture were observed on ultrasound examination (18).

### Assessment of fractures

According to interviewer-assisted, standardized, self-administered questionnaires, the history of fractures was collected. Data regarding fracture sites and the age when the fracture occurred were also collected. In this study, osteoporotic fractures were defined as low-trauma fractures that occurred at age ≥45 years due to falling from a standing height or shorter, a trip or slip, or falling out of bed (fracture of the hip, wrist, spine) (19, 20). All pathologic (due to cancer, bone tuberculosis, etc.) and traumatic fractures (defined as a fracture that resulted from a fall from higher than a standing height or an accident due to a motor vehicle collision or sports, etc.) were excluded (19, 20). We verified the fracture based on the hospital diagnosis or previous radiographic data.

### Statistical analysis

In accordance with previous studies, we performed all analyses integrally and separately for men and women because sex may be an important influencing factor for the association between NAFLD and osteoporotic fracture. Continuous variables were presented as mean ± standard deviation, and categorical variables were presented as number and proportions. Comparisons between groups were performed using chi-squared tests for categorical variables, one-way analysis of variance (ANOVA) for normally distributed continuous variables, and the Kruskal Wallis test for skewed continuous variables. Odds ratios (OR) and corresponding 95% confidence intervals (CI) for the associations between NAFLD and osteoporotic fracture were calculated using logistic regression analyses. Both unadjusted and multivariate-adjusted logistic regression analyses were performed: model 1 was adjusted for age; model 2 was model 1 plus smoking status and alcohol status; model 3 was model 2 plus physical activity (<30 min/day, 30–60 min/day, >60 min/day); model 4 was model 3 plus diabetes, hypertension, cardiovascular events, and family history of fracture; model 5 was model 4 plus waist circumference, BMI, and dyslipidemia; and model 6 was model 5 plus TG, TC, HDL-C, and LDL-C. Two-sided *P* < 0.050 was considered significant. Statistical analyses were performed using PASW Statistics 18.0 software (SPSS Inc., Chicago, IL, USA), Empower (R) (www.empowerstats.com; X&Y Solutions Inc., Boston, MA, USA), and R packages (http://www.r-project.org; The R Foundation for Statistical Computing, Vienna, Austria).

## Results

### Participant characteristics

Of the 2,659 participants included in the current analyses, 614 had NAFLD (226 men and 388 women) and 383 had fractures (96 men and 287 women). The clinical and laboratory characteristics, stratified by NAFLD status, are shown in Table 1. Compared to participants without NAFLD, participants with NAFLD had a significantly higher prevalence of fractures (13.30 vs. 18.08%; *P* = 0.003) and comorbidities, including hypertension (37.73 vs. 59.02%; *P* < 0.001), cardiovascular events (23.14 vs. 38.40%; *P* < 0.001), hypertension (39.40 vs. 62.70%; *P* < 0.001), and diabetes mellitus (13.43 vs. 24.14%; *P* < 0.001).

**Table 1:** Characteristics of participants stratified by NAFLD status.

	**Total participants** (*n* = **2,659**)			**Men** (*n* = **950**)			**Women (1,709)**		
	**Participants without NAFLD**	**Participants with NAFLD**	***p*-value**	**Participants without NAFLD**	**Participants with NAFLD**	***p*-value**	**Participants without NAFLD**	**Participants with NAFLD**	***p*-value**
*N*	2,045	614		724	226		1,321	388	
Age (years)	73.78 ± 6.17	72.42 ± 5.51	<0.001	74.71 ± 5.93	72.97 ± 5.63	<0.001	73.27 ± 6.24	72.10 ± 5.42	<0.001
Waistline (cm)	82.35 ± 8.97	86.58 ± 9.56	<0.001	85.30 ± 8.79	90.39 ± 8.94	<0.001	80.73 ± 8.65	84.31 ± 9.21	<0.001
BMI (kg/m^2^)	23.41 ± 3.40	25.26 ± 3.09	<0.001	23.45 ± 2.96	25.34 ± 2.68	<0.001	23.38 ± 3.62	25.20 ± 3.31	<0.001
Diastolic pressure	129.75 ± 15.92	132.73 ± 15.37	<0.001	130.78 ± 15.23	134.10 ± 14.53	0.004	129.19 ± 16.27	131.92 ± 15.80	0.004
Systolic pressure	79.11 ± 9.35	80.61 ± 9.14	<0.001	80.48 ± 9.52	82.46 ± 9.84	0.007	78.37 ± 9.18	79.52 ± 8.53	0.031
TC (mmol/L)	5.22 ± 0.96	5.31 ± 1.02	0.034	4.94 ± 0.90	5.05 ± 1.04	0.143	5.37 ± 0.96	5.47 ± 0.98	0.078
TG (mmol/L)	1.66 ± 0.91	2.01 ± 1.12	<0.001	1.57 ± 0.87	1.92 ± 1.04	<0.001	1.71 ± 0.93	2.07 ± 1.17	<0.001
HDL-C (mmol/L)	1.51 ± 0.39	1.42 ± 0.31	<0.001	1.39 ± 0.36	1.34 ± 0.28	0.025	1.57 ± 0.40	1.47 ± 0.31	<0.001
LDL-C (mmol/L)	2.80 ± 0.71	2.86 ± 0.75	0.134	2.64 ± 0.68	2.75 ± 0.74	0.030	2.90 ± 0.70	2.93 ± 0.75	0.431
Total hip BMD (T-score)	−1.92 ± 1.12	−1.76 ± 1.14	0.002	−1.58 ± 1.12	−1.41 ± 1.07	0.058	−2.10 ± 1.08	−1.95 ± 1.14	0.019
BMI (kg/m^2^)			<0.001			<0.001			<0.001
<18.5	81 (3.96%)	6 (0.98%)		25 (3.45%)	2 (0.88%)		56 (4.24%)	4 (1.03%)	
18.5 to 24.9	1,431 (69.98%)	292 (47.56%)		508 (70.17%)	105 (46.46%)		923 (69.87%)	187 (48.20%)	
≥24.9	533 (26.06%)	316 (51.47%)		191 (26.38%)	119 (52.65%)		342 (25.89%)	197 (50.77%)	
Loss weight >7%			<0.001			<0.001			<0.001
No	1,727 (84.49%)	269 (43.95%)		622 (86.03%)	112 (49.78%)		1,105 (83.65%)	157 (40.57%)	
Yes	317 (15.51%)	343 (56.05%)		101 (13.97%)	113 (50.22%)		216 (16.35%)	230 (59.43%)	
Smoke status			0.422			0.470			0.702
Never	1,658 (81.19%)	488 (79.74%)		367 (50.76%)	108 (48.00%)		1,291 (97.88%)	380 (98.19%)	
Ever or current	384 (18.81%)	124 (20.26%)		356 (49.24%)	117 (52.00%)		28 (2.12%)	7 (1.81%)	
Alcohol status			0.063			0.077			0.683
Never	1,714 (84.60%)	491 (81.43%)		443 (61.61%)	122 (54.95%)		1,271 (97.25%)	369 (96.85%)	
Ever or current	312 (15.40%)	112 (18.57%)		276 (38.39%)	100 (45.05%)		36 (2.75%)	12 (3.15%)	
Physical activity			0.904			0.861			0.965
<30 min/day	506 (24.98%)	156 (25.62%)		171 (23.85%)	57 (25.56%)		335 (25.59%)	99 (25.65%)	
0.5–1 h/day	779 (38.45%)	236 (38.75%)		282 (39.33%)	87 (39.01%)		497 (37.97%)	149 (38.60%)	
>1 h/day	741 (36.57%)	217 (35.63%)		264 (36.82%)	79 (35.43%)		477 (36.44%)	138 (35.75%)	
Family history of fracture			<0.001			0.023			<0.001
No	1,658 (88.38%)	444 (81.62%)		589 (91.04%)	163 (85.34%)		1,069 (86.98%)	281 (79.60%)	
Yes	218 (11.62%)	100 (18.38%)		58 (8.96%)	28 (14.66%)		160 (13.02%)	72 (20.40%)	
Diabetes			<0.001			<0.001			<0.001
No	1,766 (86.57%)	465 (75.86%)		625 (86.69%)	166 (73.45%)		1,141 (86.50%)	299 (77.26%)	
Yes	274 (13.43%)	148 (24.14%)		96 (13.31%)	60 (26.55%)		178 (13.50%)	88 (22.74%)	
Hypertension			<0.001			<0.001			<0.001
No	1,238 (60.60%)	229 (37.30%)		416 (57.54%)	70 (30.97%)		822 (62.27%)	159 (40.98%)	
Yes	805 (39.40%)	385 (62.70%)		307 (42.46%)	156 (69.03%)		498 (37.73%)	229 (59.02%)	
Cardiovascular events			<0.001			<0.001			<0.001
No	1,571 (76.86%)	377 (61.60%)		569 (78.70%)	142 (62.83%)		1,002 (75.85%)	235 (60.88%)	
Yes	473 (23.14%)	235 (38.40%)		154 (21.30%)	84 (37.17%)		319 (24.15%)	151 (39.12%)	
High TC			0.266			0.275			0.474
No	820 (41.10%)	229 (38.55%)		387 (54.20%)	110 (50.00%)		433 (33.80%)	119 (31.82%)	
Yes	1,175 (58.90%)	365 (61.45%)		327 (45.80%)	110 (50.00%)		848 (66.20%)	255 (68.18%)	
High TG			<0.001			0.001			<0.001
No	1,296 (64.96%)	303 (51.01%)		485 (67.93%)	123 (55.91%)		811 (63.31%)	180 (48.13%)	
Yes	699 (35.04%)	291 (48.99%)		229 (32.07%)	97 (44.09%)		470 (36.69%)	194 (51.87%)	
Low HDL-C			0.444			0.838			0.398
No	1,762 (88.37%)	518 (87.21%)		664 (93.13%)	204 (92.73%)		1,098 (85.71%)	314 (83.96%)	
Yes	232 (11.63%)	76 (12.79%)		49 (6.87%)	16 (7.27%)		183 (14.29%)	60 (16.04%)	
High LDL-C			0.231			0.509			0.262
No	1,236 (61.99%)	352 (59.26%)		513 (71.85%)	153 (69.55%)		723 (56.48%)	199 (53.21%)	
Yes	758 (38.01%)	242 (40.74%)		201 (28.15%)	67 (30.45%)		557 (43.52%)	175 (46.79%)	
Fracture			0.003			0.010			0.047
No	1,773 (86.70%)	503 (81.92%)		661 (91.30%)	193 (85.40%)		1,112 (84.18%)	310 (79.90%)	
Yes	272 (13.30%)	111 (18.08%)		63 (8.70%)	33 (14.60%)		209 (15.82%)	78 (20.10%)	

*Data are means ± SD or numbers (percent). P values were calculated from chi-squared test for categorical variables, the one-way ANOVA for normally distributed continuous variables, and the Kruskal Wallis test for skewed continuous variables*.

Men with NAFLD were younger (72.97 ± 5.63 vs. 74.71 ± 5.93 years; *P* < 0.001) than men without NAFLD. Men with NAFLD had a significantly larger waist circumference (90.39 ± 8.94 vs. 85.30 ± 8.79 cm; *P* < 0.001), significantly higher BMI (25.34 ± 2.68 vs. 23.45 ± 2.96 kg/m^2^; *P* < 0.001), serum TG (1.92 ± 1.04 vs. 1.57 ± 0.87 mmol/L; *P* < 0.001), and LDL-C (2.75 ± 0.74 vs. 2.64 ± 0.68 mmol/L; *P* = 0.030), and significantly lower high-density lipoprotein cholesterol (HDL-C) (1.34 ± 0.28 vs. 1.39 ± 0.36 mmol/L; *P* = 0.025) than men without NAFLD. Additionally, men with NAFLD had a higher frequency of fractures compared to men without NAFLD (14.60 vs. 8.70%; *P* = 0.010). Similarly, women with NAFLD had a significantly larger waist circumference (84.31 ± 9.21 vs. 80.73 ± 8.65 cm; *P* < 0.001), significantly higher BMI (25.20 ± 3.31 vs. 23.38 ± 3.62 kg/m^2^; *P* < 0.001) and TG (2.07 ± 1.17 vs. 1.71 ± 0.93 mmol/L; *P* < 0.001), and significantly lower HDL-C (1.47 ± 0.31 vs. 1.57 ± 0.40 mmol/L; *P* < 0.001) than women without NAFLD. Furthermore, there was a borderline difference in the prevalence of osteoporotic fractures in women with NAFLD compared with women without NAFLD (15.82 vs. 20.10%; *P* = 0.047).

### Association between NAFLD and osteoporotic fracture

Table 2 shows the association between NAFLD and osteoporotic fracture. For all participants, NAFLD was significantly associated with increased odds of osteoporotic fractures in the unadjusted model (OR, 1.45; 95% CI, 1.14–1.86; *P* = 0.003). However, after adjusting for age, smoking status, alcohol status, physical activity, diabetes, hypertension, cardiovascular events, family history of fracture, dyslipidemia, waist circumference, BMI, TG, TC, HDL-C, LDL-C, and BMD (model 7), the association was no longer significant (OR, 1.25; 95% CI, 0.93–1.68; *P* = 0.1365). A similar pattern of associations was also detected for women (unadjusted model: OR, 1.34; 95% CI, 1.00–1.79; *P* = 0.048; model 7: OR, 1.05; 95% CI, 0.74–1.48; *P* = 0.800).

**Table 2:** Association between NAFLD and osteoporotic fracture.

	**Men**		**Women**		**Total participants**	
	**OR (95%CI)**	***p-*value**	**OR (95%CI)**	***p*-value**	**OR (95%CI)**	***p*-value**
Unadjusted	1.79 (1.14, 2.82)	0.0110	1.34 (1.00, 1.79)	0.0478	1.45 (1.14, 1.86)	0.0025
Model 1	1.77 (1.13, 2.80)	0.0135	1.39 (1.04, 1.86)	0.0256	1.50 (1.17, 1.91)	0.0012
Model 2	1.69 (1.06, 2.69)	0.0271	1.39 (1.04, 1.86)	0.0282	1.47 (1.15, 1.89)	0.0022
Model 3	1.69 (1.06, 2.70)	0.0269	1.39 (1.04, 1.86)	0.0273	1.48 (1.15, 1.89)	0.0020
Model 4	1.77 (1.06, 2.97)	0.0304	1.33 (0.97, 1.82)	0.0766	1.45 (1.11, 1.90)	0.0067
Model 5	1.81 (1.02, 3.22)	0.0434	1.11 (0.78, 1.58)	0.5715	1.29 (0.95, 1.74)	0.0973
Model 6	1.81 (1.01, 3.27)	0.0477	1.15 (0.80, 1.66)	0.4494	1.33 (0.98, 1.81)	0.0700
Model 7	1.86 (1.06, 3.27)	0.0296	1.05 (0.74, 1.48)	0.7998	1.25 (0.93, 1.68)	0.1365

*Data are OR (95% CI). Model 1 was adjusted for age; Model 2 was further adjusted for smoking status and alcohol status (Never/Ever or current) based on model 1; Model 3 was further adjusted for physical activity (<30 min a day/0.5–1 h a day/>1 h a day) based on model 2; Model 4 was further adjusted for diabetes, hypertension, cardiovascular events, family history of fracture based on model 3; Model 5 was further adjusted for waistline, BMI, loss weight >7% based on model 4; Model 6 was further adjusted for serum TG, TC, HDL-c, and LDL-c based on model 5, Model 7 was further adjusted for BMD based on model 6*.

Conversely, for men, NAFLD was significantly associated with increased odds of osteoporotic fractures in the unadjusted model (OR, 1.79; 95% CI, 1.14–2.82; *P* = 0.011) and marginally associated with increased odds of osteoporotic fracture risk in model 7 (OR, 1.86; 95% CI, 1.06–3.27; *P* = 0.030).

### Association between serum cholesterol level and fracture type

Table 3 shows the association between NAFLD and osteoporotic fracture type. There was a significant association between NAFLD and lumbar fractures in all participants and in men alone, whereas no association was observed in women. Moreover, there were no significant associations between NAFLD and radius fractures, humerus fractures, or hip fractures in both men and women.

**Table 3:** Association between NAFLD and lumbar fracture, radius fracture, hip fracture and humerus fracture.

	**Men**		**Women**		**Total participants**	
	**OR (95%CI)**	***p-*value**	**OR (95%CI)**	***p*-value**	**OR (95%CI)**	***p-*value**
Lumbar fracture	2.62 (1.09, 6.32)	0.0316	1.50 (0.86, 2.59)	0.1506	1.74 (1.09, 2.76)	0.0191
Radius fracture	1.49 (0.36, 6.22)	0.5859	0.84 (0.32, 2.25)	0.7336	1.04 (0.47, 2.31)	0.9290
Hip fracture	3.12 (0.56, 17.32)	0.1937	1.09 (0.43, 2.75)	0.8608	1.43 (0.65, 3.14)	0.3718
Humerus fracture	/	/	1.45 (0.43, 4.87)	0.5524	2.41 (0.80, 7.28)	0.1195

*Data are OR (95% CI). Adjusted for age, smoking status and alcohol status (never/ever or current), physical activity (<30 min a day/0.5–1 h a day/>1 h a day), diabetes, hypertension, cardiovascular events, family history of fracture, loss weight >7%, waistline, BMI, and BMD*.

### Stratified analysis of the association between NAFLD and osteoporotic fractures for different dyslipidemia statuses

We examined whether dyslipidemia status affected the association between NAFLD and osteoporotic fractures. As shown in Table 4, we observed a differential association between NAFLD and osteoporotic fractures between men with and without high TG, low HDL-C, and high LDL-C. After adjusting for age, smoking status, alcohol status, physical activity, diabetes, hypertension, cardiovascular events, family history of fractures, dyslipidemia, waist circumference, BMI, TG, TC, HDL-C, LDL-C, and BMD, no significant interactive effects were detected (interaction *P* > 0.05 for all).

**Table 4:** Effect of dyslipidemia on the association between NAFLD and osteoporotic fracture.

	**Men**			**Women**		
	**OR (95%CI)**	***p*-value**	*p*-value^*^	**OR (95%CI)**	***p*-value**	*p* value^*^
**HIGH TC**						
No	2.10 (0.90, 4.86)	0.0842	0.3583	1.38 (0.72, 2.66)	0.3313	0.6184
Yes	0.89 (0.33, 2.40)	0.8109		0.90 (0.57, 1.42)	0.6492	
**HIGH TG**						
No	2.61 (1.15, 5.90)	0.0213	0.0826	0.92 (0.54, 1.58)	0.7719	0.5267
Yes	0.74 (0.27, 2.02)	0.5601		1.28 (0.76, 2.16)	0.3576	
**LOW HDL-C**						
No	1.88 (1.01, 3.52)	0.0472	/	0.88 (0.58, 1.32	0.5386	0.0934
Yes	/	/		3.82 (1.42, 10.27)	0.0078	
**HIGH LDL-C**						
No	2.13 (1.01, 4.47)	0.0467	0.7024	1.11 (0.66, 1.85)	0.6957	0.3079
Yes	1.01 (0.32, 3.19)	0.9925		1.05 (0.61, 1.80)	0.8661	

* * P value for test of interaction*.

*Adjusted for age, smoking status and alcohol status (never/ever or current), physical activity (<30 min a day/0.5–1 h a day/>1 h a day), diabetes, hypertension, cardiovascular events, family history of fracture, loss weight >7%, waistline, BMI, and BMD*.

## Discussion

In the present cross-sectional study, NAFLD was significantly associated with risk of osteoporotic fractures in men 55 years or older; however, no association was observed in women. A further stratified analysis showed that there was a significant association between NAFLD and risk of osteoporotic fractures in men without dyslipidemia.

According to the unadjusted model, NAFLD was significantly associated with increased odds of osteoporotic fractures for women. However, after adjusting for age, smoking status, alcohol status, physical activity, diabetes, hypertension, cardiovascular events, family history of fracture, dyslipidemia, waist circumference, BMI, TG, TC, HDL-C, LDL-C, and BMD, the association was no longer significant. Moreover, according to a dyslipidemia status subgroup analysis, a significant association between NAFLD and risk of osteoporotic fractures only remained for men without dyslipidemia status, which might imply a true effect of age.

Previous studies have primarily focused on the relationship between NAFLD and BMD and have reported contradictory results. For example, many observational studies reported that there was a significant association between NAFLD and osteopenia/osteoporosis (21, 22). Conversely, a meta-analysis of five cross-sectional studies showed no significant difference in BMD between patients with and without NAFLD (*n* = 1,276 participants, 638 cases of NAFLD); however, obesity and insulin resistance may have affected this association (23). On the contrary, a prospective cohort study consisting of 1,659 participants found that there was a significant inverse association between liver fat content and lumbar spine, hip, and whole-body BMD (β = −0.123, *P* = 0.001; β = −0.101, *P* = 0.008; β = −0.130, *P* < 0.001, respectively) and osteocalcin (β = −0.116, *P* = 0.001) for men; however, no associations were observed for postmenopausal women (β = −0.068, *P* = 0.068 for lumbar spine BMD; β = −0.029, *P* = 0.429 for hip BMD; β = −0.041, *P* < 0.224 for whole-body BMD; β = −0.025, *P* < 0.498 for osteocalcin) (21). Moreover, a large, retrospective, cross-sectional study comprising 6,634 participants showed that there was a significant negative association between femoral neck BMD and NAFLD for men (β = −0.013, *P* = 0.029), but that there was a positive association between lumbar spine BMD and NAFLD for postmenopausal women (β = 0.022, *P* = 0.005) (24).

Our analyses showed similar but generally stronger associations between NAFLD and osteoporotic fracture risk compared to those indicated by the only available cross-sectional study of this issue that found that NAFLD may have increased the OR of osteoporotic fracture risk for men (14). This study suggested that there may be other factors that interfere with the association between NAFLD and risk of osteoporotic fractures. However, the authors did not exclude middle-aged participants (especially postmenopausal women); therefore, some confounding factors, such as menopause, may have affected the real association between NAFLD and risk of osteoporotic fractures, which may have reduced the strength of their conclusions. Moreover, the authors did not examine whether dyslipidemia status affected the association. To our knowledge, our study is the first observational study involving older men and women (age ≥55 years) that examined the association between NAFLD and risk of osteoporotic fracture and that evaluated whether dyslipidemia status affects this association. Therefore, our study reported several new findings.

**Figure 1:**
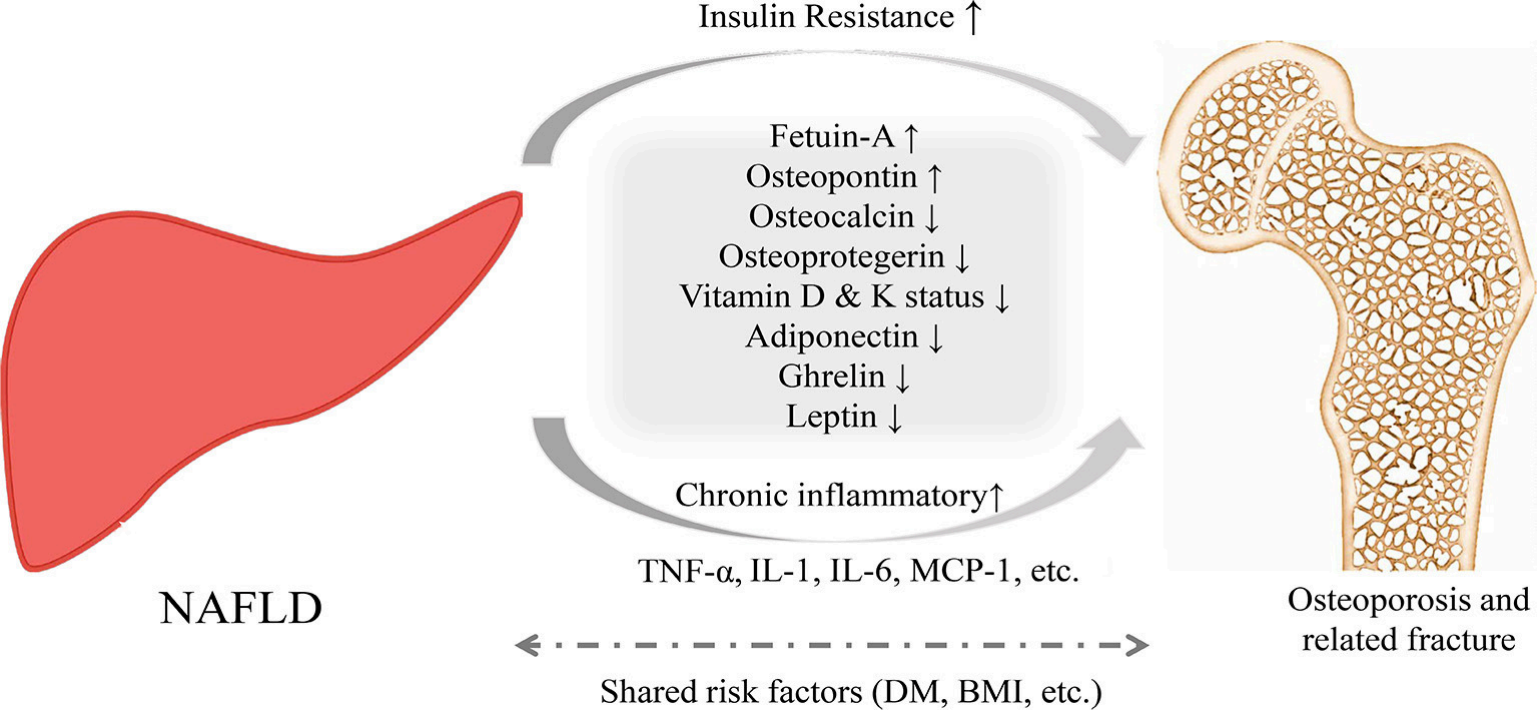
Proposed pathophysiological mechanisms for the relationship between NAFLD and osteoporosis and related fractures.

To date, the pathophysiological links between NAFLD and osteoporosis and related fractures are unclear (13, 25– 27). Therefore, exploring potential mechanisms that link NAFLD and osteoporosis is very important and may potentially lead to additional and novel prevention and treatment strategies (Figure 1).

For example, insulin resistance is considered to be a major risk factor for NAFLD, and this may also be observed in patients with lower BMI or normal glucose tolerance. Accumulation of intrahepatic lipids is associated with a decrease in insulin sensitivity in the liver, bone, muscle, and fat (28). Previous studies reported that higher fasting serum insulin and insulin resistance may be associated with higher risk of lower BMD and lower femoral neck strength (29, 30). However, this association remains controversial (31, 32). Experimental studies showed that in rats consuming a high-fat diet, development of insulin resistance could decrease osteoblast proliferation and differentiation and increase osteoblast apoptosis, resulting in decreased jaw bone density (33).

Chronic inflammation may be another important cross-talk mechanism linking NAFLD and osteopenia/osteoporosis. For example, higher concentrations of interleukin-1, interleukin-6, and tumor necrosis factor-α were found with several liver diseases, such as cirrhosis, hepatitis, and alcoholic liver disease. Moreover, these inflammatory cytokines may enhance the activity of osteoclasts, inhibit the apoptosis of osteoclasts, and stimulate osteoclastogenesis (34–37).

Vitamin D is a fat-soluble vitamin that is involved in bone-related metabolism (38–40), and 25-hydroxyvitamin D, which is the most important circulating and storing metabolite of vitamin D, is produced in the liver. Previous studies have shown that vitamin D deficiency has had a role in the pathogenesis of both NAFLD and osteoporosis (41, 42). Further well-designed, randomized clinical trials are needed to investigate the potential for vitamin D supplementation in NAFLD patients to reduce the risk of lower BMD and fractures.

Osteocalcin, a marker of bone formation, is mainly expressed by osteoblasts. Osteocalcin^−/−^ knockout mice have exhibited increased fat mass and insulin resistance and decreased expression of insulin target genes and adiponectin genes (43). Additionally, several epidemiological studies investigated the association between osteocalcin and NAFLD (44–47). In a Chinese cohort study, an inverse correlation was found between serum osteocalcin level and the scale of NAFLD (*r* = −0.150, *P* < 0.010) (45). Other studies also confirmed this inverse relationship between osteocalcin and NAFLD in both male and female participants (44, 46).

Osteoprotegerin (OPG), an important coordinator in the balance between bone formation and bone resorption, is associated with obesity and insulin resistance (48). The possible role of OPG in the pathogenesis of NAFLD is controversial. Epidemiological studies reported that serum OPG in patients with NAFLD was much lower than that in patients without NAFLD (49, 50). However, other studies have yielded contradictory results (51). The role of OPG in relation to NAFLD and BMD needs further investigation.

Our study had several limitations. First, the current study had a cross-sectional design, which cannot draw a causal inference. Further well-designed, prospective cohort studies are needed to investigate a potential causal relationship. Second, recall bias is innate and there is the possibility for confusion regarding data from participants' medication history and dietary records. Third, our study used verified, interviewer-assisted, standardized, self-administered questionnaires for the assessment of fractures, thereby possibly missing vertebral fractures. Fourth, risk of osteoporotic fractures is associated with many factors, such as lifestyle, disease status, metabolic factors, inflammatory states, and genetic factors (3–5, 52, 53). Although wide epidemiologic and clinical covariables were included in the adjustment, we could not exclude the possibility of residual confounding variables, such as bone markers, vitamin D/calcium, decreased glucose levels, and muscle strength, in the analyses. Therefore, additional well-designed and stratified cohort studies that include sufficient controls and account for confounding factors are needed to elucidate the link between NAFLD and the risk of osteoporotic fractures. Fifth, our study was underpowered for the assessment of NAFLD. Liver biopsy is the gold standard for the diagnosis of NAFLD. In our study, the diagnosis of NAFLD was based on ultrasonic examination, which may have underestimated the incidence of NAFLD. However, due to its invasiveness and the potential for complications, liver biopsy is not commonly used in epidemiological studies or in the clinic. Ultrasonography is a noninvasive and simple tool for the diagnosis of NAFLD. According to previous epidemiological studies, ultrasonic examination was the most commonly used assessment for NAFLD. Finally, although a wide variety of epidemiologic and clinical covariates were included in the adjustment, we cannot exclude the possibility of residual confounders in the analyses. Therefore, our results should be interpreted with caution.

In conclusion, our study demonstrated that there was a significant association between NAFLD and osteoporotic fracture risk in older Chinese men, and particularly in men without dyslipidemia. Additional well-designed and stratified cohort studies with a wide range of controls for confounding factors are required to elucidate the link between NAFLD and the risk of osteoporotic fractures.

## Author contributions

YW, CH, and YC: contributed to the study design; YW, GW, RZ, WZ, and SL: performed data collection, interpretation, and analysis; CH and YC: performed critical review. All authors performed data collection, case diagnoses, and confirmation of case diagnoses.

### Conflict of interest statement

The authors declare that the research was conducted in the absence of any commercial or financial relationships that could be construed as a potential conflict of interest.

## Abbreviations

95% CI: 95% confidence interval
BMD: bone mineral density
BMI: body mass index
HDL-C: high-density lipoprotein cholesterol
LDL-C: low-density lipoprotein cholesterol
NAFLD: nonalcoholic fatty liver disease
OR: odds ratio
TC: total cholesterol
TG: serum triglycerides.
