# Supplementary figures and images for: Association of Nonalcoholic Fatty Liver Disease With Osteoporotic Fractures: A Cross-Sectional Retrospective Study of Chinese Individuals
Source: Front Endocrinol (Lausanne). 2018 Jul 23;9:408. doi: 10.3389/fendo.2018.00408 (PMC6064874; doi:10.3389/fendo.2018.00408)

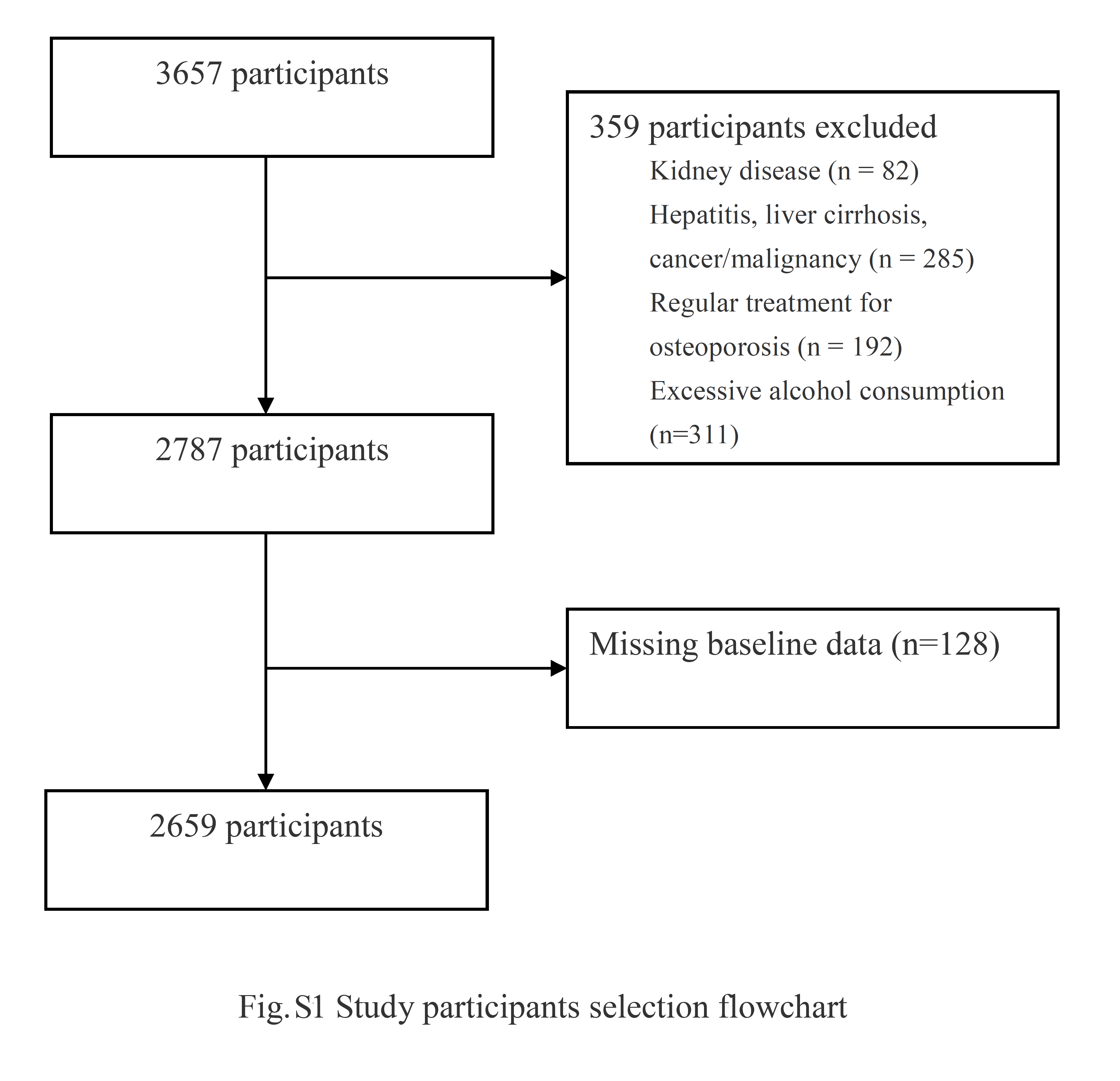

Supplement: Supplementary file 1 [file Image_1.TIF]
